# Occult Hepatitis B Virus Infection in Patients With Chronic Hepatitis C Treated With Antiviral Therapy

**DOI:** 10.5812/hepatmon.7292

**Published:** 2012-11-30

**Authors:** Gian Paolo Caviglia, Maria Lorena Abate, Paola Manzini, Franca Danielle, Alessia Ciancio, Chiara Rosso, Antonella Olivero, Rinaldo Pellicano, Giovanni Antonio Touscoz, Antonina Smedile, Mario Rizzetto

**Affiliations:** 1 Department of Internal Medicine, University of Turin, Turin, Italy; 2 Blood Bank, San Giovanni Battista University Hospital (Molinette), Turin, Italy; 3 Department of Gastroenterology and Hepatology, San Giovanni Battista University Hospital (Molinette), Turin, Italy

**Keywords:** Hepatitis C, Chronic, Hepatitis B Virus, Hepatitis C, Infection

## Abstract

**Background:**

Occult hepatitis B virus infection (OBI) is defined as the presence of hepatitis B virus (HBV) DNA in the liver and/or in the serum of patients with negative results of hepatitis B s antigen (HBsAg) test with or without serological markers of previous viral exposure. The impact of OBI in patients with chronic hepatitis C (CHC) is still unclear.

**Objectives:**

The Aim of this study was to assess OBI prevalence and its potential implications on treatment outcome in a cohort of patients with CHC underwent standard antiviral therapy.

**Patients and Methods:**

Baseline serum samples from 137 HBsAg-negative CHC patients treated with pegylated-interferon and ribavirin (73 Responders/74 Non Responders),were retrospectively analyzed for HBV status.

**Results:**

Seventy-three patients (53.3%) showed markers of previous exposure to HBV. HBV DNA was detected in 2 of 137 serum samples (1.5%), both carrying HBV antibodies. Liver biopsies and post-therapy sera were available for 35 patients (12 Responders/23 Non Responders). HBV DNA sequences were found in 13 of 35 specimens (37.1%), all of patients with HBV DNA negativity in basal and post-therapy serum samples. Among OBI-positive patients, 5 (38.5%) carried serological markers of HBV infection. Regarding therapy outcome, in the OBI-positive group there were 5 of 13 (38.5%) sustained virological responders (SVR) compared to 7 of 22 (31.8%) in the OBI-negative one.

**Conclusions:**

Despite the high prevalence rate of liver HBV DNA in patients with CHC, SVR was not affected by occult HBV infection.

## 1. Background

Occult hepatitis B virus infection (OBI) is defined as the presence of hepatitis B virus (HBV) DNA in the liver of patients with negative results of hepatitis B s antigen (HBsAg) test with or without serological markers of previous viral exposure (1-3). In these patients, the lack of circulating HBsAg may be due to rearrangements in the HBV genome which interfere with gene expression or lead to the production of an antigenically modified S protein (4-6). The molecular basis of occult HBV infection is related to the long-lasting persistence in the nuclei of hepatocytes of the viral covalently-closed-circular DNA (cccDNA) (7). Almost all OBI cases are infected with replication-competent HBV showing strong suppression of replication and gene expression, probably due to host immune-surveillance and epigenetic factors (8). On the basis of HBV antibodies profile, OBI may be distinguished in seropositive- and seronegative-OBI; the former is positive for hepatitis B core and/or s antibodies, the latter is negative for all markers of HBV infection aside very low amount of HBV DNA (< 200 IU/ml) (8). Seronegative-OBI cases may have either progressively lost HBV specific antibodies after the resolution of an acute infection or, theoretically, had negative results of tests from the beginning of infection, similar to what has been observed in the woodchuck model of hepadnavirus infection with the woodchuck hepatitis virus (9), where a low-dose infection was insufficient to allow the maturation of an antiviral protective memory response (10). Although OBI status is significantly associated with the presence of antibodies to HBV (3), the analysis of liver DNA extracts represents the gold standard for occult HBV evaluation (8). Hence, serum analysis must be taken into account only in the absence of liver specimens. In any case, it is strongly recommended to use a highly sensitive nested polymerase chain reaction (PCR) or real time PCR with oligonucleotide primers specific for different HBV genomic regions and complementary to highly conserved nucleotide sequences (11). Occult HBV infection has been found with a high prevalence in patients with chronic hepatitis C (CHC), probably because both HBV and hepatitis C virus (HCV) share the same parenteral way of transmission. In particular, HBV DNA is detectable in about one-third of CHC patients with negative results of HBsAg test in the Mediterranean basin (3).

## 2. Objectives

Since it is unclear the impact of OBI in patients with CHC, the aim of this study was to evaluate OBI prevalence and its possible implications on antiviral therapy outcome in a cohort of patients treated for CHC.

## 3. Patients and Methods

One hundred thirty seven CHC patients with negative results of HBsAg test, treated with pegylated interferon (peg-IFN) and ribavirin, were included in the study. The study was performed according to the 1975 Declaration of Helsinki. Written informed consent was obtained from all patients prior to recruitment. The main demographic characteristics are reported in Table 1. Baseline serum samples were analyzed for HBV antibodies (anti-HBs, anti-HBc and anti-HBe) with standard assays. For HBV DNA detection, in baseline and selected six month follow-up serum samples, viral nucleic acids were purified from a 400 µl specimen by automated procedure (EZ1 Virus Mini Kit v.2, BioRobot EZ1 Advanced, QIAGEN). Liver biopsies were available for a subgroup of 35 serum matched patients. Frozen liver specimens (10-12 mg) were disrupted in TRIZOL by a rotor-stator homogenizer. After purification, liver DNA concentration and quality were assessed with spectrophotometer (NanoDrop ND 1000, NanoDrop Technologies). All serum and liver nucleic acids extracted were tested for HBV DNA by four parallel nested-PCRs to detect HBV S, Core, and Pol and X sequences (detection limit 5 IU/mL). PCR primers were complementary to highly conserved nucleotide sequences of HBV genome (8). Two rounds of amplification, 35 cycles each, were performed using HotStartTaq Polymerase (Qiagen, Germany). Appropriate negative and positive controls were included in each PCR experiment. To check for false negatives a parallel PCR for beta-globin gene was performed. In addition, direct sequencing of amplicons obtained by nested PCR was performed to confirm the specificity of the reactions. Samples turned positive for at least two gene targets were scored as OBI-positive according to Taormina expert meeting statements (8). Statistical analysis was performed using Student’s t test or Mann-Whitney U test to compare continuous variables and Fisher’s exact test to analyze categorical data. All P values are two-tailed; a P value below 0.05 was considered statistically significant.

**Table 1:** Baseline Characteristics of Patients

Characteristic	All Patients	Liver Biopsy Subgroup
No. of patients	137	35
Sex (M/F)	89/48	22/13
Age, y, range	48.0 (29-69)	48.4 (29-68)
HCV genotype 1, No. (%)	78 (56.9%)	28 (80%)
Therapy outcome R vs. NR	73 vs. 64	12 vs. 23

Abbreviations: M, male; F, female; HCV, hepatitis C virus; R, responders; NR, non-responders.

## 4. Results

Among the 137 HCV patients with negative results of HBsAg test, HBV antibodies were detected in 73 (53.3%) (Table 2), while serum HBV DNA was detected only in 2 (1.5%), both positive for the four HBV genomic regions were examined (S, Core, Pol, X). Each of them carried markers of previous HBV exposure (one positive for anti-HBs, anti-HBc and anti-HBe, the other positive for both anti-HBc and anti-HBe). One patient was responder to HCV therapy while the other was not. Because of low number of cases with positive results of HBV DNA test in their sera, no statistical correlation was detected. Liver biopsies were available for 35 of 137 patients. Intra-hepatic HBV DNA was detected in 13 of 35 patients (37.1%) with nested PCR for at least two different HBV genomic regions, but none of the corresponding basal or follow-up serum samples tested positive. In detail, two cases had positive results of all four HBV regions examined; six had positive results for three regions (S, Core and Pol) and 5 for two regions (three for S and Core; one for Core and Pol; one for S and Pol). Eight of 13 patients with occult HBV infection had negative results for serum anti-HBV antibodies test, whereas the remaining five were seropositive for at least one marker of previous HBV exposure (one for anti-HBc, two for anti-HBc and anti-HBs, one for anti-HBc and anti-HBe, one for anti-HBs). Their characteristics are reported in Table 3. There was no statistical difference between OBI-positive and OBI-negative patients regarding sex, age, HBV antibodies, aminotransferase levels, liver fibrosis and basal HCV viral load. Patients who responded to HCV antiviral treatment were equally distributed between HBV DNA-positive and -negative patients: 5/13 (38.5%) versus 7/22 (31.8%; P = 0.726), respectively.

**Table 2:** Serum Markers of Previous HBV Exposure

	All Patients	Liver Biopsy Subgroup
**HBV markers positive, No. (%)**	73 (53.3%)	19 (54.3%)
Anti-HBc positive	25 (18.3%)	7 (20%)
**Anti-HBc/anti-HBs positive**	24 (17.5%)	7 (20%)
Anti-HBc/anti-HBe positive	9 (6.6%)	2 (5.7%)
Anti-HBc/anti-HBs/anti-HBe positive	13 (9.5%)	1 (2.9%)
Anti-HBs positive	2 (1.5%)	2 (5.7%)

Abbreviations: HBV, hepatitis B virus; anti-HBc, antibodies to hepatitis B core antigen; anti-HBs, antibodies to hepatitis B surface antigen; anti-HBe, antibodies to hepatitis B e antigen.

**Table 3:** Characteristics of Patients With Liver Biopsy (n = 35) According to OBI Status

	OBI positive	OBI negative	*P* value
**No. of patients**	13 (37.1%)	22 (62.9%)	
**Sex (M/F)**	10/3	12/10	0.282
**Age, y, range**	50.1 (31-68)	47.4 (29-66)	0.681
**AST, IU/L, mean ± SD**	82 ± 35	63 ± 32	0.112
**ALT, IU/L, mean ± SD**	136 ± 84	102 ± 64	0.183
**Ishak histology fibrosis score, mean ± SD**	2.69 ± 0,63	2.86 ±1.46	0.863
**Ishak histology activity score, mean ± SD**	4.69 ± 1.25	4.45 ± 2.22	0.483
**HCV genotype 1, No. (%)**	11 (85%)	17 (77%)	0.689
**Basal HCV Viral load, IU/ml, median**	2.0 × 106	1.6 × 106	0.441
**Therapy outcome R vs. NR, % R**	5 vs. 8 (38.5%)	7 vs. 15 (31.8%)	0.726
**HBV markers positive, No. (%)**	5 (38.5%)	14 (63.6%)	0.179
Anti-HBc positive	1 (7.7%)	6 (27.3%)	
Anti-HBc/anti-HBs positive	2 (15.4%)	5 (22.7%)	
Anti-HBc/anti-HBe positive	1 (7.7%)	1 (4.6%)	
Anti-HBc/anti-HBs/anti-HBe positive	0	1 (4.6%)	
Anti-HBs positive	1 (7.7%)	1 (4.6%)	

Abbreviations: OBI, occult hepatitis B virus infection; M, male; F, female; AST, aspartate aminotransferase; ALT, alanine aminotransferase; HCV, hepatitis C virus; R, responders; NR, non-responders; HBV, hepatitis B virus; anti-HBc, antibodies to hepatitis B core antigen; anti-HBs, antibodies to hepatitis B surface antigen; anti-HBe, antibodies to hepatitis B e antigen.

## 5. Discussion

In the present study serum HBV DNA was detected in two of 137 (1.5%) CHC patients with negative results of HBsAg test. A higher prevalence rate was found in liver, where HBV sequences were detected in 13 of 35 (37.1%) specimens. Among the 13 OBI-positive patients, only five (38.5%) carried serological markers of HBV infection. The prevalence rate of OBI in patients with CHC patients reported in the literature varies greatly, ranging from 0% to 52% (12, 13). This wide range might be linked to the geographical distribution of HBV infection (14) as well as to the sensitivity of the method used to detect HBV DNA, including PCR primer selection (15), and OBI definition (13). Examination of liver DNA extracts is the “gold standard” for OBI testing, but it is not always applicable in clinical practice (2, 13). In fact, HBV DNA is detected in liver tissue specimens of patients with serum HBV DNA but is often undetectable in serum of patients with intra-hepatic HBV DNA (16). For this reason, if a liver biopsy specimen is not available, analysis of serum samples should be performed with a highly sensitive and specific approach based on nested PCR or real time PCR. Moreover, it is recommended to perform serum HBV detection at different time points because the analysis of only one sample, drawn before therapy, could not be sufficient to detect OBI if virus replication is intermittent (17). Many studies reported a higher prevalence of HBV-DNA detection in anti-HBc-positive than in anti-HBc-negative patients (3,18, 19). In our cohort, similarly to what reported by several authors (10, 15, 20, 21), since 8 of 13 patients with positive results of OBI test were seronegative, OBI was not associated with the presence of anti-HBc antibodies. Previous reports (12, 15) showed that serum HCV-RNA load was significantly higher in CHC OBI-positive patients than in negative ones. In our study, in agreement with other groups (22-25), no statistically significant differences were found. To evaluate whether occult HBV infection contributes to liver damage in CHC patients, we compared histological scores according to the presence of HBV DNA. As previously reported by other authors (20, 24, 25) we did not find any association between OBI and severity of chronic liver disease. However, other studies (2, 15, 16) found that severe lesions were more common in CHC patients with positive results of OBI test compared to those with HCV infection alone. Chen et al. (23) reported that patients with both OBI and HCV infection had lower ALT levels, liver histology activity index and fibrosis scores than those with HCV monoinfection. The clinical impact of OBI on anti-HCV therapy outcome is still controversial. Preliminary studies, focusing on the treatment response to IFN monotherapy, suggested an association between the presence of OBI and a lower virological response rate (12, 16). Although some recent studies (20, 24-26) with peg-IFN plus ribavirin therapy, showed that the presence of OBI had no or minimal effect on the outcome, while others did not confirm these data (15). In the present study, patients responders to HCV antiviral treatment were equally distributed between patients with positive and negative results of HBV DNA testing (38.5% versus 31.8%, respectively) confirming that the rate of sustained virological response to peg-IFN plus ribavirin combination therapy is similar in the two groups. Finally, a recent study (27) showed a strong association between the presence of OBI in patients with CHC and the development of hepatocellular carcinoma (HCC) when compared to patients with monoinfected HCV. Moreover, OBI was strongly associated with liver cancer independently of age, sex, HCV co-infection and cirrhosis. These findings suggested that OBI might contribute to hepatocyte transformation, playing a direct oncogenic role through both its integration into the host genome and a maintained transcriptional activity, allowing the synthesis of proteins with potential pro-oncogenic properties. In the present study, it was not possible to draw such conclusion because none of the 35 patients developed HCC in the two years after the end of treatment. Nevertheless, a closer follow-up is recommended in patients with positive results of OBI test for the potential risk of HCC development.
